# Epidemiology and molecular characterization of methicillin-resistant *Staphylococcus aureus* nasal carriage isolates from bovines

**DOI:** 10.1186/1746-6148-10-153

**Published:** 2014-07-10

**Authors:** Stéphanie Nemeghaire, M Angeles Argudín, Freddy Haesebrouck, Patrick Butaye

**Affiliations:** 1 Department of General Bacteriology, Veterinary and Agrochemical Research centre, Groeselenbergstraat 99, B-1180 Ukkel, Belgium; 2 Department of Pathology, Bacteriology and Avian diseases, Faculty of Veterinary Medicine, Ghent University, Salisburylaan 133, 9820 Merelbeke, Belgium

**Keywords:** Nasal carriage, Bovine, Epidemiology, Molecular characterization, Antimicrobial resistance

## Abstract

**Background:**

*Staphylococcus aureus* is a common bacterium usually found on skin and mucous membranes of warm blooded animals. Resistance in *S. aureus* has been increasingly reported though depending on the clonal lineage. Indeed, while hospital acquired (HA)-methicillin resistant *S. aureus* (MRSA) are typically multi-resistant, community associated (CA)-MRSA are by large more susceptible to many antibiotics. Although *S. aureus* isolated from animals are often susceptible to most antibiotics, multi-resistant livestock associated (LA)-MRSA have been recovered from bovine mastitis.

In this study, we investigated the prevalence and types of MRSA present in the nose of healthy bovines of different age groups and rearing practices. Since no validated methods for MRSA isolation from nasal swabs were available, we compared two isolation methods. Molecular characterization was performed by means of *spa*-typing, MLST, SCC*mec* typing and microarray analysis for the detection of antimicrobial resistance and virulence genes.

**Results:**

MRSA between herd prevalence in bovines was estimated at 19.8%. There was a marked difference between rearing practices with 9.9%, 10.2% and 46.1% of the dairy, beef and veal calve farms respectively being MRSA positive. No significant difference was observed between both isolation methods tested. Most isolates were ST398 *spa* type t011 or closely related *spa* types. Few ST239 *spa* type t037 and t388 and ST8 *spa* type t121 were also found. SCC*mec* types carried by these strains were mainly type IV(2B), IV(2B&5) and type V. Type III and non-typeable SCC*mec* were recovered to a lesser extent. All isolates were multi-resistant to at least two antimicrobials in addition to the expected cefoxitin and penicillin resistance, with an average of resistance to 9.5 different antimicrobials. Isolates selected for microarray analysis carried a broad range of antimicrobial resistance and virulence genes.

**Conclusion:**

MRSA were mainly present in veal farms, compared to the lower prevalence in dairy or beef farms. Multi-resistance in these strains was high. Though mainly CC398 *spa* t011 was found, the genetic diversity was higher than what was found for pigs in Belgium. CC8 strains, a typically human lineage but also recently found also in association with bovines, has been retrieved here also.

## Background

*Staphylococcus aureus* is a common facultative pathogenic bacterium that has long been recognized as a burden in both human and veterinary medicine. Indeed, *S. aureus* has been shown to be responsible of various infections such as clinical and subclinical bovine mastitis [1,2], wound infections in horses [3-5], dogs [6] and wild animals such as hedgehogs [7]. Furthermore, *S. aureus* is well known to harbour resistance to antimicrobial agents which may lead to complications in the treatment of its infections [8] and increase the cost of treatments [9]. One of these antimicrobial resistances is encoded by the *mecA* gene conferring resistance to almost all β-lactams including methicillin, oxacillin and cephalosporins. Though first considered not causing many infections [10], MRSA have more recently been shown to be present in 10% of Belgian farms suffering from *S. aureus* bovine mastitis [11]. Livestock associated (LA)-MRSA was first described in pigs in 2005 and humans in close contact with pigs in the Nederland [12] and in France [13]. This particular clone belonging to the clonal complex (CC)398 was later encountered in many healthy animals such as pigs [14], horses [15], bovines [16] and poultry [17-19]. This clonal complex is composed of different closely related *spa* types [20] and cannot be typed by pulsed field gel electrophoresis using SmaI digestion [21].

Although MRSA in bovines and in cases of bovine mastitis are well documented, information about the prevalence of *S. aureus* and MRSA in healthy bovines is lacking.

For international comparisons, a standardized isolation method is necessary. The European Food Safety Authority (EFSA) [22] has proposed a standardized protocol for the isolation of MRSA from dust samples obtained from pig farms. However, this protocol was estimated not to be very sensitive in a study in poultry in 2011 [19].

The aim of this study was to determine the prevalence and epidemiology of MRSA in bovines and compare the EFSA proposed isolation method with an alternative enrichment method in order to determine whether there were differences between the two methods in this population.

## Methods

### Sampling and isolation method

Four hundred and thirty-two farms were examined during the national survey on bovine MRSA in Belgium 2012. These farms were randomly selected from the Sanitel database. Of these, 141 were dairy farms, 187 farms reared beef cattle and 104 reared veal calves. Per farm, nose swabs were taken from 20 animals and pooled. Sampling was performed by the Belgian Federal Agency for the Safety of the Food Chain. Ethics approval was not required for this study under Belgian regulations, as taking a nasal swab does not cause pain, distress or lasting harm.

The first method was the standard method proposed by ESFA [22], MRSA was isolated using 100 mL Mueller-Hinton (MH) broth (Becton Dickinson, US) supplemented with NaCl (6.5%) and incubated at 37°C for 20 to 24 h. One ml of this broth was added to Tryptic Soy Broth (TSB) supplemented with cefoxitin (3.5 mg/l) and aztreonam (75 mg/l) and incubated overnight at 37°C. Ten μl of this broth was plated on MRSA selective plate, MRSA-ID (bioMérieux, Marcy-l’Etoile, France), and incubated 48 hours at 37°C. At both 24 and 48 hours, plates were inspected and suspected colonies were purified on Columbia agar plates with 5% sheep blood (CSB) (Bio Rad Laboratories, Nazareth Eke, Belgium) and incubated overnight at 37°C. Since this isolation method includes two enrichment steps, it is referred in this study as double broth enrichment method (DBEM).

The alternative method was applied to 106 farms and differed from the DBEM protocol by the omission of the second enrichment in antibiotic supplemented broth. For this reason, this second isolation methods is referred as single broth enrichment method (SBEM).

### DNA extraction, MRSA identification and characterization

DNA was extracted as previously described [3]. MRSA identification and *mecA* gene detection was performed using a triplex PCR previously published [23].

A PCR allowing the detection of the clonal complex (CC) 398 was performed on all MRSA following a protocol previously described by Stegger *et al.*[24]. MRSA isolates that were negative in the CC398 PCR were subjected to multi-locus sequence typing (MLST) [25]. Sequences of seven internal fragments were then compared to the international database [26] to obtain the sequence type. Strains were further characterised by *spa*–typing, as previously described [27]. The resulting *spa type*s were assigned by using the Ridom StaphType software [28]. Clustering of *spa types* was performed using the algorithm Based Upon Repeat Pattern (BURP) available in the Ridom StaphType software. Staphylococcal cassette chromosome *mec* (SCC*mec*) types were determined by the means of two multiplex PCRs (M-PCRs) designed for the detection of the *mec*-complex and *ccr*-complex [29]. Appropriate control strains were used.

### Antimicrobial susceptibility testing

Antimicrobial resistance was determined using a micro broth dilution method (Sensititre, Trek Diagnostic Systems, Magellan Biosciences, Ohio, USA). The Minimal inhibitory concentrations (MIC) of 19 antimicrobials (penicillin, cefoxitin, kanamycin, streptomycin, gentamicin, erythromycin, clindamycin, quinupristin/dalfopristin, linezolid, tiamulin, chloramphenicol, rifampicin, ciprofloxacin, fusidic acid, tetracycline, trimethoprim, sulfamethoxazole, vancomycin, and mupirocin) were determined as previously described [20]. The MIC values were interpreted using the European Committee on Antimicrobial Susceptibility Testing (EUCAST) epidemiological cut-offs (ECOFF) for *S. aureus*. Data from the EUCAST MIC distribution website was last accessed November 6, 2013 [30].

### DNA microarray-based typing and detection of resistance and virulence genes

Fourteen isolates were selected based on the different antimicrobial resistance phenotypes for detection of resistance and virulence genes by the mean of microarray analysis. Microarray analysis was performed on these strains using the Identibac *S. aureus* Genotyping DNA Microarray (Alere Technologies GmbH, Köln, Germany) according to the manufacturer’s instructions. The DNA microarray covers 333 oligonucleotide probes, detecting resistance and virulence genes. A full list including primer and probe sequences is available online [31].

### Statistical analysis

The number of resistant isolates was counted and resistance percentages were calculated. The Cohen's kappa coefficient was calculated in order to compare both isolation methods. Cohen’s kappa coefficient was interpreted according to Landis & Koch [32]. This analysis includes the first 106 farms. DBEM is considered as the Gold standard while SBEM is the one under estimation. Apparent prevalence, true prevalence, Cohen’s Kappa coefficient, sensitivity, specificity, positive predictive value (PPV) and negative predictive value (NPV) of both methods were also calculated using a previously described formulae [33] and Win Episcope 2.2 (Clive, United Kingdom). Pearson chi square and Fisher’s exact test were computed using IMB SPSS Statistics^®^ Version 20.0.

## Results

### Prevalence

Overall, and using the official DBEM, 81 farms (19.8%, 95% confidence interval (CI) [15.1% - 22.4%]) were positive for MRSA (Table 1). There was no significant difference between the between-herd prevalence of dairy and beef farm (Fisher’s exact test; p = 0.549) while there was a significant difference between the between-herd prevalence of dairy and veal calf farms (Fisher’s exact test; p < 0.001) and between the between-herd prevalence of beef and veal calf farms (Fisher’s exact test; p < 0.001). Fourteen dairy farms (9.9%, 95% CI [5.0% - 14.9%]), 19 farms holding beefs (10.2%, 95% CI [5.8% - 14.5%]) and 48 farms rearing veal calves (46.1%, 95% CI [36.6% - 55.7%]) were found positive for MRSA.

**Table 1:** Total number of MRSA isolates corresponding to the different genotypes recovered and separated by farm types

	**Prevalence (%)**	**95% CI**	**MLST**			***spa *** **types**										**SCC** ***mec***				
			**8**	**239**	**398**	**t011**	**t037**	**t121**	**t388**	**t1451**	**t1456**	**t1985**	**t3423**	**t6228**	**NT**	**III (3A)**	**IV (2B)**	**IV (2B&5)**	**V (5C2)**	**NT**
DF	9.93	[4.99 - 14.9]	0	2	12	8	1	0	1	0	1	0	0	2	1	1	4	0	6	3
VF	46.15	[36.6 - 55.7]	1	0	18	16	0	1	0	0	1	1	0	0	0	1	12	0	3	3
BF	10.16	[5.83 - 14.5]	0	0	48	40	0	0	0	3	1	3	1	0	0	0	30	11	7	0
Total	18.75	[15.1 - 22.4]	1	2	78	64	1	1	1	3	3	4	1	2	1	2	46	11	16	6

BF, Beef farm; DF, dairy farm; MLST, Multi locus sequence typing; NT, non-typeable; VF, veal farm.

### Comparison of isolation methods

Comparisons were performed on 106 samples. Using both isolation methods (Table 2), 34 (32.1%, 95% CI [23.2% - 41.1%]) farms out of 106 tested were found to be positive. Among these positive farms recovered, nine farms were detected positive with the SBEM but not with the DBEM and conversely, nine other farms were detected positive with the DBEM but not with the SBEM. Kappa agreement coefficient (k) was 0.61 which indicates a substantial agreement between both methods. There was no significant difference between the prevalence of these methods (Fisher’s exact test; p =0.597). Specificity, positive predictive value and negative predictive value were likewise identical (Table 3).

**Table 2:** **Comparison of the number of methicillin-resistant *Staphylococcus aureus *** **isolates detected using Double Broth Enrichment Method (DBEM) or Single Broth Enrichment Method (SBEM)**

		**DBEM**		**Total**
		**Positive**	**Negative**	
SBEM	Positive	25	9	34
	Negative	9	63	72
Total		34	72	106

**Table 3:** Comparison of the test evaluation of both isolation methods

	**DBEM (%)**	**SBEM (%)**	**95% CI lower limit**	**95% CI upper limit**
Apparent prevalence	32.1	32.1	23.2	41.0
True prevalence	40.6	40.6	31.2	49.9
Sensitivity	79.1	79.1	66.9	91.2
Specificity	100.0	100.0	100.0	100.0
Predictive value positive	100.0	100.0	100.0	100.0
Predictive value negative	87.5	87.5	79.9	95.1

CI, Confidence interval; DBEM, Double broth enrichment method; SBEM, Single broth enrichment method.

### MLST, *spa*- and SCC*mec* typing

Among 81 MRSA isolates recovered, seventy-eight (96.3%) were positive in the CC398 PCR. The three other isolates were ST8 and two ST239, as demonstrated by MLST. All calf isolates were CC398. The ST8 was recovered from beef cattle and both ST239 isolates were isolated from dairy farms (Table 1).

**Figure 1:**
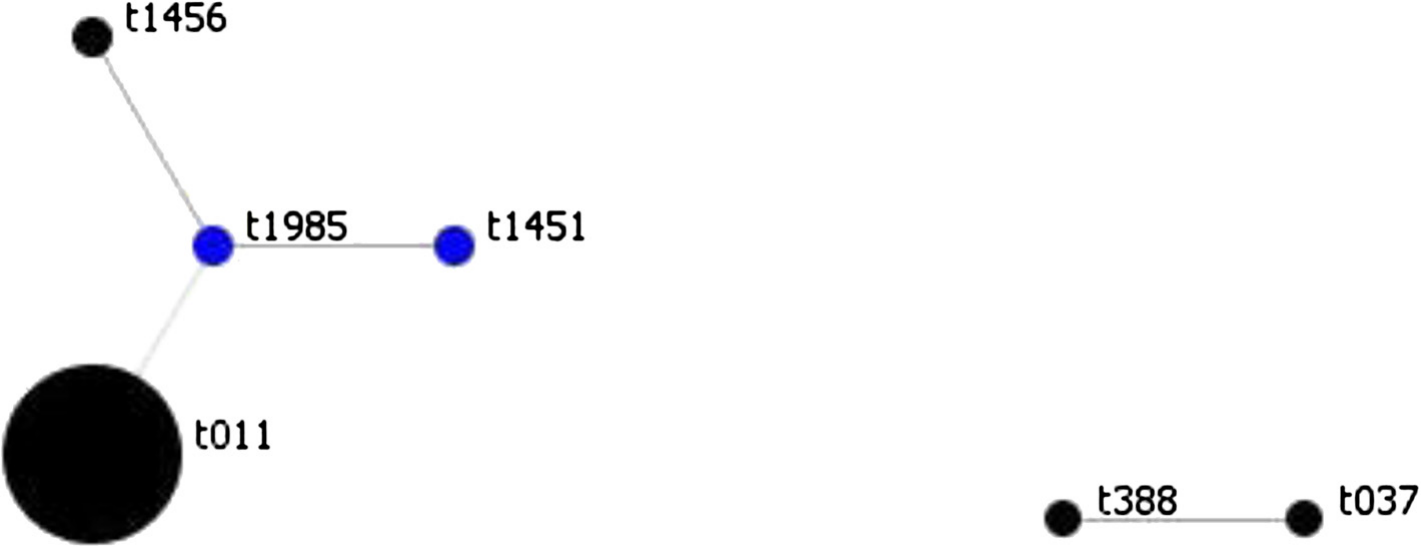
**Clustering of *spa types *** **performed using Based Upon Repeat Pattern (BURP) algorithm.**

Ten different *spa* types were identified. Sixty-four (79.0%) were *spa* type t011. Other *spa* types recovered were t037 (n = 1), t121 (n = 1), t388 (n = 1), t1451 (n = 3), t1456 (n = 3), t1985 (n = 4), t3423 (n = 1), t6228 (n = 2) and a non-typeable *spa* type. Two clusters were distinguished using the BURP algorithm (Figure 1). The first cluster, including 92% of all isolates and 44% of all *spa* types, grouped the *spa* types t011, t1451, t1456 and t1985. The second cluster, which included 3% of all isolates and 22% of all *spa* types, grouped the *spa* types t037 and t388. A singleton was also detected with the *spa*-type t121. The remaining *spa* types t3423 and t6228 could not be aligned by the software. All t011 and closely related *spa*-type isolates were associated to CC398. MRSA *spa* type t121 was associated to MLST type ST8, while t388 and t037 to ST239. The MRSA t011 and closely related strains were isolated from veal (n = 47), beef (n = 18) and dairy farms (n = 9). The t3423 and t6228 MRSA were isolated from veal (n = 1) and dairy farms (n = 2). The t037, t388 and the non-typable *spa* type MRSA were recovered from dairy farms and the t121 MRSA was recovered in beef farm (Table 1).

Forty-four (54.3%) isolates carried SCC*mec* type IV(2B) and nine (11.1%) SCC*mec* type IV(2B&5). Sixteen (19.8%) isolates carried SCC*mec* type V(5C2) and two (2.5%) SCC*mec* type III(3A). Ten (12.3%) isolates showed only the *mecA* gene but no *ccr* complex was detected with the PCR. These were thus considered non-typeable using these M-PCRs. SCC*mec* type IV (2B and/or 2B&5) were found in isolates from veal (n = 37), beef (n = 12) and dairy farms (n = 4). SCC*mec* type V were also found in the three age groups with seven being found in isolates from veal, six from dairy and three from beef cattle. Type III cassette were found in from dairy (n = 1) and beef cattle (n = 1). The non-typeable SCC*mec* was detected in isolates from veal calves (n = 4), dairy (n = 3) and beef (n = 3) cattle. Additionally to the type IV(2B) (n = 43), IV(2B&5) (n = 9), V (n = 16) and non-typeable (n = 8) SCC*mec*, CC398 MRSA isolated also carried the type III (n = 2) SCC*mec.* Both t121 and the non-typeable *spa* type carried SCC*mec* type IV(2b) and *spa* types t388 and t037 carried a non typeable SCC*mec*.

### Antimicrobial resistance

All isolates were resistant to cefoxitin and penicillin as expected. More than 90% of the isolates were resistant to tetracycline (96.3%) and trimethoprim (95.1%). A high prevalence of resistance was also observed to clindamycin (86.4%), erythromycin (86.4%), kanamycin (80.2%) and gentamicin (76.5%). More than half of the isolates were also resistant to streptomycin (58.0%). Lower resistance levels were detected to fusidic acid (27.2%), sulfamethoxazole (25.9%), quinupristin/dalfopristin (23.5%), tiamulin (17.3%), ciprofloxacin (16.0%), chloramphenicol (12.3%), rifampicin (12.3%) and mupirocin (9.9%). No resistance was observed to linezolid and vancomycin (Table 4). All isolates were at least resistant to two more antimicrobials in addition to cefoxitin and penicillin. More than 50% of the isolates were resistant to nine or more different antimicrobials. Two isolates were resistant to 16 different antimicrobials, remaining susceptible only to ciprofloxacin, linezolid and vancomycin. The isolates resistant to 15 (n = 3) or 16 (n = 2) antibiotics were all CC398 *spa* type t011. Two of these isolates carried a non-typable cassette and three carried SCC*mec* type IV (2B). These originated from veal (n = 3) and beef cattle (n = 2). The one isolate resistant to 14 antibiotics was a CC398 *spa* type t6228 strain carrying SCC*mec* type V. The one isolate resistant to only four antibiotics was a CC398 *spa* type t1456 strain carrying SCC*mec* type V and originated from a farm holding beef cattle. Isolates that were resistant to five (n = 1) and six (n = 5) antimicrobials were CC398 *spa* type t011 carrying SCC*mec* type V (n = 3) and IV (2B; n = 1) or t1985 (n = 2). These isolates were isolated from veal calves (n = 3), dairy (n = 1) and beef cattle (n = 2). The ST8 isolate was resistant to seven different antimicrobials and both ST239 isolates were resistant to nine different antimicrobials. There were no significant differences in resistance prevalence between veal calves, dairy and beef cattle.

**Table 4:** **MIC distribution in methicillin-resistant *S. aureus *** **isolates from bovines**

**Antimicrobials**	**% of isolates with MIC (mg/l) of**																**% R**
	**≤0.016**	**0.03**	**0.06**	**0.12**	**0.25**	**0.5**	**1**	**2**	**4**	**8**	**16**	**32**	**64**	**128**	**256**	**512**	
CHL									4.9	46.9	35.8	2.5	8.6	1.2			12.3
CIP					18.5	27.2	9.9	2.5	2.5	11.1	28.4						16.0
CLI				12.3	1.2	1.2	1.2	0.0	0.0	84.0							86.4
ERY					3.7	7.4	2.5	0.0	2.5	2.5	81.5						86.4
FOX						0.0	0.0	0.0	0.0	3.7	18.5	77.8					100.0
FUS						72.8	12.3	2.5	9.9	2.5							27.2
GEN							22.2	1.2	3.7	7.4	21.0	44.4					76.5
KAN									16.0	3.7	2.5	2.5	9.9	65.4			80.2
LZD							23.5	75.3	1.2	0.0							0.0
MUP						86.4	3.7	3.7	0.0	0.0	0.0	0.0	0.0	0.0	6.2		9.9
PEN				0.0	0.0	0.0	1.2	8.6	90.1								100.0
RIF	86.4	1.2	0.0	1.2	0.0	1.2	9.9										12.3
SMX													70.4	3.7	11.1	14.8	25.9
STR									14.8	22.2	4.9	9.9	48.1				58.0
SYN						32.1	44.4	8.6	8.6	6.2							23.5
TET						2.5	1.2	0.0	0.0	0.0	1.2	95.1					96.3
TIA						75.3	7.4	0.0	0.0	17.3							17.3
TMP								4.9	3.7	1.2	0.0	1.2	88.9				95.1
VAN							87.7	12.3	0.0	0.0	0.0						0.0

CHL, chloramphenicol; CIP, ciprofloxacin; CLI, clindamycin; ERY, erythromycin; FOX, cefoxitin FUS, fusidic acid; GEN, gentamicin; KAN, kanamycin; LZD, linezolid; MIC, minimal inhibitory concentration; MUP, mupirocin; PEN, penicillin; R, resistance; RIF, rifampicin; SMX, sulfamethoxazole; STR, streptomycin; SYN, quinupristin/dalfopristin; TET, tetracyclin; TIA, tiamulin; TMP, trimethoprim; VAN,vancomycin.

Empty boxes indicate the concentration values that were not tested. Values in grey boxes indicate MIC higher than the concentration tested.

The bold lines indicate epidemiological cut-off values for *S. aureus*. MIC values were interpreted using the EUCAST clinical breakpoints/epidemiological cut-offs [1].

### Microarray typing for resistance and virulence gene detection

Most genes were homogeneously distributed in all isolates, including typical *S. aureus* species marker and regulatory genes (23S-rRNA, *gapA*, *katA*, *coA*, *nuc*, *spa*, *sbi*, *sarA*, *saeS, vraS*), the accessory gene regulator *agrI*, haemolysins (*hla*, *hld*), genes encoding leukocidins (*lukS-*F, *hlgA*, *lukX*, *lukY*-variant 1), proteases (*aur*, *sspA*, *sspB*, *sspP*), the biofilm production genes of the *icaACD* operon, adhesion factors (*bbp*, *cflA*, *cflB*, *ebh*, *ebpS*, *eno*, *fib*, *fnbA*, *fnbB*, *map*, *sdrC*, *sdrD*, *vwb*) immune-evasion factors (*isaB*, *isdA, hysA1, hysA2*), a putative transport protein (*lmrP*), a site specific deoxyribonuclease subunit X (*hsdSx*), and staphylococcal superantigen-like proteins from the vSaα genomic islands [*setB1*, *setB2*, *setB3*, *setC*, *ssl1* (*set6*), *ssl2* (*set7*), *ssl4* (*set9*/*ssl4*), *ssl5* (*set3*/*ssl5*), *ssl7* (*set1*/*ssl7*) and *ssl10* (*set4*/*ssl10*)].

All isolates were penicillin resistant and carried the *bla* operon (*blaZ, blaI,* and *blaR*) encoding for penicillin-ampicillin resistance. All isolates, except the tetracycline sensitive one, carried the tetracycline resistance gene *tetM.* Additionnaly to *tetM*, isolates harbouring SCC*mec* type V and a non-typeable isolate carried also the tetracycline resistance gene *tetK*. Six erythromycin resistant isolates out of 11 carried the *ermC* gene. Eight gentamicin resistance isolates out of the nine tested showed the aminoglycoside adenyl‒/phosphotransferase encoding gene *aacA-aphD*. Eight kanamycin resistant isolates out of 11 carried the *aadD* aminoglycoside resistance gene and one additionally carried aminoglycoside phosphotransferase *aphA3*. One of the two chloramphenicol resistant isolate carried the *cat* (*pMC524*) gene encoding for chloramphenicol acetyltransferase. All isolates carried the putative transport protein *sdrM*. The metallothiol transferase (*fosB*) gene encoding fosfomycin resistance was detected in both non CC398 isolates. Furthermore, most isolates carried an intact beta-haemolysin gene (*hlb*), except the ST239 isolate which harboured the *hlb* gene truncated after the probable insertion of the immune-evasion phage-borne genes *sak* (staphylokinase) and *scn* (staphylococcal complement inhibitor).

## Discussion

In this study, we found an estimated MRSA prevalence of 19.8% in bovine farms in Belgium. As found in The Netherlands [16] and a small former Belgian study [34], the prevalence in veal calve farms sampled using nasal swabs was much higher than in dairy farms or farms holding beef cattle. In contrast, the prevalence found at veal calve farms was lower than in these previous studies. In the Netherlands, MRSA prevalence in veal calve farms was estimated at 88% [16] while the small scale Belgian study estimated a prevalence of 64% [34]. In this study, swabs were pooled according to EFSA recommendations [35], while in the other two studies, ten to 25 individual samples per farm were analyzed. The lower prevalence in our study may thus be explained by the differences in sampling since it has been shown that a culture of pooled swabs may have a lower sensitivity than separated swabs culture [36]. Compared to other livestock animals, the estimated prevalence in bovines is much higher than that in poultry (0.8%) [20] but lower than that in pigs (68%) [14].

The isolation method used throughout the study (the DBEM) was the method recommended by EFSA and the European reference laboratory. During the comparison, nine isolates were detected while using the DBEM and not with the alternative method. Conversely nine isolates were detected using the SBEM while not with the DBEM. These isolates have probably been lost during the second enrichment step since this includes antimicrobials. However, as shown for samples from poultry [19], representing a low prevalence population, the second enrichment method does not make any statistical difference. Therefore we recommend for future European surveillances to use the SBEM on nasal swabs.

Most isolates were typical LA-MRSA CC398 *spa* type t011 or closely related *spa* types. Three other MLST types were recovered: ST8 *spa* type t121 and ST239 *spa* type t037 and t388. Those three types are usually identified among hospital-acquired (HA)-MRSA. However, while MRSA *spa* type t121 was uncommonly found in Belgian hospitals [37] it has been commonly recovered in hospitals in Europe and in the United States [38]. This *spa* type has also been found in bulk tank milk in the United States [39]. MRSA ST239 *spa type* t388 and t037 are widespread HA-MRSA found in Europe, Asia and America [40]. A MRSA ST239 t037 was also isolated from poultry in 2011 [19]. The recovery of these HA-MRSA from livestock indicates that one should remain vigilant to the evolution of MRSA in animals. Though not investigated in his study, these strains in general carry a multitude of virulence genes on mobile genetic elements. Transfer of these virulence genes to LA-MRSA CC398 would have a huge impact on the importance of this clone for human health and its epidemiology in animals.

The diversity of *spa* types seen in this study in bovines was larger than what has been found previously in pigs in Belgium, where only *spa* type t011 and t034 were found [14,41]. In bovines, at least seven different *spa* types were recovered among the MRSA CC398 isolates. It has been concluded previously that the length of the *spa* gene sequence may depend on the fact that isolates are methicillin resistance or not, or on the source of *S. aureus* isolation [42]. Since our isolates were all methicillin resistant and *spa*-types were found to be closely related, the hypothesis of a possible host adaptation is supported. Also the diversity of the SCC*mec* types in isolates from cattle seems to be larger than what is found in pigs, however the two predominant types are the same, SCC*mec* type IV and SCC*mec* type V. Surprisingly, two isolates carried SCC*mec* type III. This type is typically associated with HA-MRSA [43] and has also been found extensively in *Staphylococcus* spp. other than *S. aureus* from animals. SCC*mec* type III has been described before in ST398, but these were in fact variant SCC*mec* type V [44,45]. Next to this, six isolates carried a non-typeable SCC*mec* cassette. Further studies are needed to be able to estimate the plasticity of the SCC*mec*, since this may be of importance to the epidemiology of MRSA in livestock and humans.

The level of multi-resistance is extremely high since it accounts for an average of 9.5 different antimicrobials. Most isolates were resistant to tetracycline and trimethoprim additionally to the expected resistance to cefoxitin and penicillin. In this study two CC398 isolates were found to be susceptible to tetracycline while tetracycline susceptible strains are only very rarely found in CC398 MRSA [46]. The prevalence of erythromycin, clindamycin, kanamycin and gentamicin resistance in this collection is extremely high compared to what has been found in strains from other origins in Belgium [14,34]. The isolate with the lowest level of multi-resistance was resistant to two additional antimicrobials. Two isolates were resistant to sixteen antimicrobials out of nineteen tested excluding ciprofloxacin, linezolid and vancomycin, three antimicrobials that are used as a last resort in the treatment of MRSA infections in humans.

The staphylokinase (*sak*) and the staphylococcal complement inhibitor (*scn*) genes carried on bacteriophage of the φ3 family were found only on the ST239 isolate but not on the most typical LA-MRSA. Since this bacteriophage family are commonly found in human isolates but few in isolates from animals or humans in contact with pigs [47,48], this might indicate a human to animal transmission of non CC398 isolates. Most resistance and virulence gene detected were homogeneously distributed amongst isolates except for the macrolide/lincosamide resistance encoding gene *erm(C)* which was found in more than half of the erythromycin resistant isolates and the fosfomycin resistance gene *fosB* which was detected in two non CC398 isolates. Additionally to resistance genes, virulence factors such as leukocidins, proteases, staphylococcal superantigen like proteins, haemolysins genes, genes involved in adhesion and immune-evasion were also found in all isolates tested by micro-array. Our results are similar to those of a previous micro-array based study performed in Germany [49] on *S. aureus* isolates from cattle in which leukicidins, haemolysins and enterotoxin genes were detected in most isolates. According to this study, staphylokinase (*sak*) was also absent in most of our isolates except for the ST239 isolate. However, while in the German study toxic shock syndrome toxins, were demonstrated, the *tst-*1 gene was not detected in our isolates. Additionally, genes encoding adhesion factors including the bone sialoprotein-binding protein (*bbp*), the cell wall associated fibronectin‒binding protein (*ebh*), the fibrinogen binding protein (*fib*), the fibronectin‒binding protein (*fnbB*) and the major histocompatibility complex class II analog protein (*map*) were detected in all isolates. These genes were also found in MRSA isolates from Sahiwal cattle with mastitis in India [50]. Our results show that, although our isolates came from apparently healthy carrier animals, MRSA in bovines may carry a broad range of different resistance genes and virulence factor that might play an important role in the pathogenicity of the bacteria.

## Conclusion

In conclusion, MRSA were found in bovines in different rearing practices. Estimated prevalence was, however, lower in nasal isolates from dairy and beef cows than from veal calves. No significant difference was observed between both isolation methods tested. The diversity of strains was larger than what was seen in pigs. Indeed, more different *spa*-types were recovered in bovine’s isolates than in pigs. Additionally, the diversity in SCC*mec* cassettes in CC398 was shown not to be limited to the types IV and V but included also type III and a non-typeable cassette. A high level of multi-resistance was found and a broad range of antimicrobial resistance and virulence genes was detected though animals sampled were apparently healthy.

## Abbreviations

CC: Clonal complex; CHL: Chloramphenicol; CI: Confidence interval; CIP: Ciprofloxacin; CLI: Clindamycin; CSB: Columbia sheep blood; DBEM: Double broth enrichment method; ECOFF: Epidemiological cut-offs; EFSA: European Food Safety Authority; ERY: Erythromycin; EUCAST: European committee on antimicrobial susceptibility Testing; FASFC: Federal Agency for the Safety of the Food Chain; FOX: Cefoxitin; FUS: Fusidic acid; GEN: Gentamicin; HA: Hospital-acquired; k: Kappa agreement coefficient; KAN: Kanamycin; LA: Livestock-associated; LZD: Linezolid; MH: Mueller-Hinton; MIC: Minimal inhibitory concentration; MLST: Multi-locus sequence typing; MUP: Mupirocin; M-PCRs: Multiplex PCRs; MRSA: Methicillin resistant *Staphylococcus aureus*; NT: Non-typable; PEN: Penicillin; R: Resistance; RIF: Rifampicin; SBEM: Single broth enrichment method, SCC*mec*, Staphylococcal cassette chromosome *mec*; SMX: Sulfamethoxazole; ST: Sequence type; STR: Streptomycin; SYN: Quinupristin/dalfopristin; TET: Tetracyclin; TIA: Tiamulin; TMP: Trimethoprim; TSB: Tryptic Soy Broth; VAN: Vancomycin.

## Competing interest

The authors declare that they have no competing interests.

## Authors’ contributions

SN performed isolation and identification of isolates, molecular typing and susceptibility testing, analyzed the data and drafted the manuscript. MAA, PB and FH participated in the design of the study provided scientific advice and helped with editing and revision of the manuscript. All authors read and approved the final manuscript.
